# Enhanced Sensitivity in Optical Sensors through Self-Image Theory and Graphene Oxide Coating

**DOI:** 10.3390/s24030891

**Published:** 2024-01-30

**Authors:** Cristina Cunha, Catarina Monteiro, António Vaz, Susana Silva, Orlando Frazão, Susana Novais

**Affiliations:** 1 INESC TEC—Institute for Systems and Computer Engineering, Technology and Science, 4150-179 Porto, Portugal; cristina.c.cunha@inesctec.pt (C.C.); catarina.s.monteiro@inesctec.pt (C.M.); antonio.v.rodrigues@inesctec.pt (A.V.); susana.o.silva@inesctec.pt (S.S.); orlando.frazao@inesctec.pt (O.F.); 2 Department of Physics and Astronomy, Faculty of Sciences, University of Porto, 4150-179 Porto, Portugal

**Keywords:** glucose detection, graphene oxide, multiphysics comsol, self-image point

## Abstract

This paper presents an approach to enhancing sensitivity in optical sensors by integrating self-image theory and graphene oxide coating. The sensor is specifically engineered to quantitatively assess glucose concentrations in aqueous solutions that simulate the spectrum of glucose levels typically encountered in human saliva. Prior to sensor fabrication, the theoretical self-image points were rigorously validated using Multiphysics COMSOL 6.0 software. Subsequently, the sensor was fabricated to a length corresponding to the second self-image point (29.12 mm) and coated with an 80 µm/mL graphene oxide film using the Layer-by-Layer technique. The sensor characterization in refractive index demonstrated a wavelength sensitivity of 200 ± 6 nm/RIU. Comparative evaluations of uncoated and graphene oxide-coated sensors applied to measure glucose in solutions ranging from 25 to 200 mg/dL showed an eightfold sensitivity improvement with one bilayer of Polyethyleneimine/graphene. The final graphene oxide-based sensor exhibited a sensitivity of 10.403 ± 0.004 pm/(mg/dL) and demonstrated stability with a low standard deviation of 0.46 pm/min and a maximum theoretical resolution of 1.90 mg/dL.

## 1. Introduction

Nowadays, optical fiber sensors (OFS) are widely applied across various domains for detecting and monitoring diverse parameters, including physical, chemical, and biological [1]. Moreover, these sensors offer various advantages, making them a very attractive topic for research and development within the scientific community. Some of these benefits include compact size, flexibility, affordability, high sensitivity, remote sensing capabilities, and immunity to electromagnetic interference compared to conventional electronic devices [2]. Specifically, over recent decades, there has been a continuous interest in refractometric-based sensors due to their capacity for easy ambient monitoring across various fields [3]. These sensors bring forth several advantages, such as label-free and real-time detection, as well as precise measurements. Coupled with the advantages previously mentioned regarding OFS, refractometry based on OFS presents an engaging and interesting option for a wide spectrum of applications. These applications span both industrial sectors, such as the food and beverage industry, environmental monitoring, and healthcare, as well as scientific domains [4,5,6]. Moreover, the relationship between refractive index (RI) and the concentration of certain substances has been studied and discussed for several years now and plays a crucial role in translating RI detection to the corresponding concentration of the analyte, such as glucose [7,8,9]. Within the biomedical industry, glucose monitoring is a real-life application worth paying attention to. Fast and accurate detection of glucose concentration holds immense importance in various sectors such as disease diagnosis, clinical analysis, biotechnology, and quality control within the food industry [10,11]. Monitoring glucose levels is crucial for maintaining human health, as abnormal levels can lead to diverse pathological conditions, such as diabetes, hypoglycemia, and hyperglycemia. Hypoglycemia manifests when blood glucose concentration falls below the normal range (between 80 and 120 mg/dL when fasting), while hyperglycemia is characterized by elevated glucose levels. Normally, diabetes is indicated when fasting blood glucose surpasses 180 mg/dL. Continuous efforts within this research community are dedicated to developing a variety of glucose sensors, including probes for glucose detection based on optical fiber sensing [6,12,13]. Most glucometers are tested on blood samples; however, previous statistical studies suggest a strong correlation between glucose salivary levels and blood levels [14]. Hence, salivary techniques can be an attractive alternative for measuring glucose levels since they can be easily applied as a noninvasive method.

In recent years, graphene and graphene-based materials have been consistently investigated as promising sensing materials [15]. While the production of graphene still involves time-consuming and expensive processes, the emergence of graphene-based materials offers a viable alternative for various sensing applications. Notably, graphene oxide (GO) exhibits a high surface area ratio, excellent biocompatibility, and cost-effectiveness in its fabrication and production. These attributes position GO as a favorable choice for a broad spectrum of sensing applications, including analytes such as gases, biomolecules, and chemical species [16,17,18].

In this paper, an optimized coreless silica fiber (CSF) tip based on the self-imaging phenomenon and graphene oxide coating is presented for sensitivity enhancement. The aim is to establish a proof-of-concept regarding the applicability of these types of sensors to measure glucose concentrations in aqueous solutions, mimicking the range typically found in human saliva.

## 2. Materials and Methods

### 2.1. Sensor Design and Principle Operation

**Figure 1:**
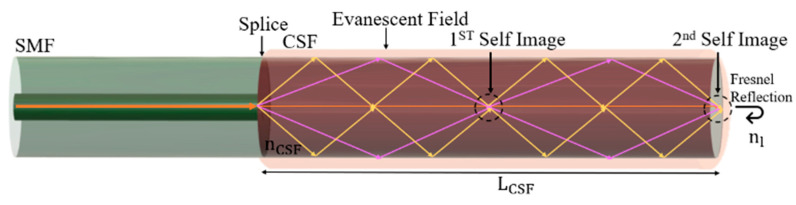
Sensor structure design, where *L_CSF_* corresponds to the CSF length—image not to scale.

The sensor design comprises a CSF (FG125LA, Thorlabs, Newton, NJ, USA) section spliced into a single-mode fiber (SMF, SM28, Thorlabs, Newton, NJ, USA), as illustrated in Figure 1. The principle of operation of the sensor consists of the coupling of light from the SMF into the CSF, inducing the excitation of multiple modes, each with distinct propagation constants. This process generates an interference pattern sensitive to external perturbations. Finally, when light reaches the CSF-external medium interface, it undergoes Fresnel reflection due to the refractive index difference between the two media, resulting in a portion of the light being reflected.

Immersing the CSF in a liquid induces the effective RI of the cladding modes to coincide with the RI of the surrounding medium. Consequently, this narrows the RI difference between the core and cladding, resulting in increased penetration of the evanescent wave generated at the CSF-liquid interface. The increase in the penetration depth of the evanescent field in the surrounding medium heightens the sensitivity of the interferometer to external changes. Moreover, as the modes interfere constructively and destructively along the fiber, it leads to the periodic formation of self-image points. These self-image points correspond to exact replicas of the input field at the CSF/external medium interface and correspond to lower loss points. Their position, *L_CSF_*, can be determined by the following equation [19]:

(1) $$L_{CSF}=p\frac{n_{CSF}D_{CSF}^{2}}{\lambda_{0}}$$

where *p* corresponds to the self-image index; *n_CSF_* is the CSF effective RI; *D_CSF_* is the CSF diameter; and *λ*_0_ is the interference wavelength. Considering the CSF from Thorlabs (FG125LA, Newton, NJ, USA) with the following characteristics: *n_CSF_* = 1.4440 RIU, *D_CSF_* = 125 µm, and *λ*_0_ = 1550 nm, and Equation (1), it is possible to determine the theoretical location of the self-image points. Table 1 presents the *L_CSF_* for different *p* indexes.

When looking at the self-image indexes, it is possible to divide them into the odd and even numbers, which respectively represent half-phase (π) and full-phase (2π) wave propagation through the fiber. This difference in the phase of the electromagnetic wave leads to differences between the odd and even numbers of self-image indices regarding coupling efficiency. As noted by Mohammed in [20], a full-phase wave leads to an enhancement in the coupling efficiency and consequently boosts the sensor performance. Therefore, the second and fourth self-image points offer better coupling efficiency when compared with the first and third self-images.

On the other hand, differences among the odd or even numbers regarding the sensitivity of a sensor are negligible. Previously, this has been experimentally verified in previous works [13,21], particularly in the context of RI sensing. This is due to the fact that alterations in the analyte RI affect the normalized frequency and propagation constant rather than the length of the coreless portion of the fiber. Thus, the effective mode of the RI remains unaffected by CSF length.

In respect of the spectral response, both theoretically and experimentally, it is expected that the second and fourth replicas of the input field exhibit a more well-defined focus point. This results in a narrower spectral response, in contrast to the first and third replicas, as presented by Guzmán-Sepúlveda [22].

### 2.2. COMSOL Multiphysics Simulation

To simulate the phenomenon of self-imaging, COMSOL Multiphysics 6.0 software was utilized, employing a CSF tip with a length mirroring the actual sensor design at 29.12 mm. As mentioned before, typically, the second and fourth self-imaging points are preferable for achieving high-quality self-imaging. However, the fourth self-image requires a longer CSF length, making it more challenging to manage. Therefore, the simulation focuses on observing the first and second self-image points.

The simulation was conducted using the wave-optics module and the beam propagation method. The electric field along the longitudinal axis was also scrutinized. More specifically, the model utilizes the Electromagnetic Waves, Beam Envelopes interface, employing a unidirectional formulation to simulate the propagation of light from a SMF to a CSF tip. Instead of a 3D model, it was decided to simulate a 2D space as it requires less computer storage, the meshing process is also simplified, and consequently, the simulation time decreases. The unidirectional formulation is applied due to the integration of single-layer anti-reflective coatings on all surfaces to suppress reflections. Additionally, the geometry is surrounded by a Perfect Match Layer (PML) to absorb ongoing waves, and an analyte surround is also added to mimic the actual environment of the tip. The thickness of the PML was 10 µm, as suggested by COMSOL and verified in [23]. Moreover, two ports are added at the beginning of the SMF, the input light, and at the end of the CSF, where the excitation is concluded. A transition boundary was added between the SMF and the CSF, with an anti-reflective coating to prevent reflections from the CSF to the SMF.

Table 2 outlines the RI and geometry settings employed in the 2D design within COMSOL.

The parameters presented in Table 2 were determined by taking into consideration the information from Thorlabs optical fiber datasheets (SMF28 and LA125, Thorlabs, USA) and the optical source used in the practical experiment to better replicate the real-life setting of the sensor.

**Figure 2:**
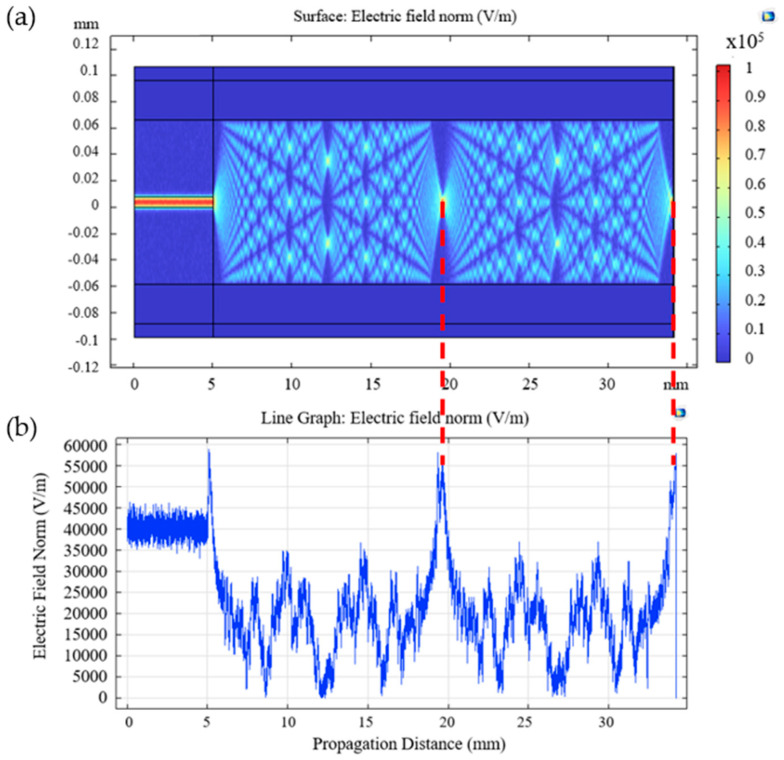
(**a**) Light propagation; (**b**) electric field distribution (longitudinal) for a CSF tip with a length of 29.12 mm.

Figure 2 shows the results derived from the simulation. Figure 2a displays the interference pattern and the self-imaging points. As expected, it is possible to observe that the light is confined in the SMF until it reaches the CSF interface, where it expands, and the interference pattern is formed. The self-image points, corresponding to the bright points observed in the pattern, are situated at 14.56 mm and 29.12 mm, right at the end of the tip, correlating with the theoretical calculations.

Figure 2b presents the distribution of the electric field, revealing two distinct peaks resulting from the electromagnetic beam being focused on the self-image points. The study of the electric field along the longitudinal axis of the CSF portion is useful to corroborate the results obtained in the simulation of light propagation in the CSF. In this case, the location of the maximum peaks of the electric field corresponds to the exact location of the self-image points that are observed in Figure 2a. However, even in the maximum peaks, differences between them can exist, as reported by Sepúlveda [22]. It is shown that the amplitude of the electric field regarding the first self-image is significantly lower than that of the fourth self-image. Moreover, and still following the previous work, the results hint at a typical filter-like spectral response. This means that only one wavelength can produce a replica of the input field on the x-axis for a fixed propagation distance.

### 2.3. Fabrication of Coreless Silica Tips

The fabricated tips have a length of L_CSF_ = 29.12 mm, so the second self-image point corresponds to the end of the fiber. First, the CSF cladding is removed by immersing the fiber in a solution of acetone for two minutes, and then by removing it with fiber strippers. After the cladding removal, the CSF was cleaved by a machine (FC-6RS, Sumitomo Electric, Osaka, Japan) and spliced into a SMF that will serve as the waveguide for the input light. The alignment was made in automatic mode by the cleaved machine since the diameter of both fibers is the same. Therefore, it is ensured that the core of the SMF is centered on the position of the CSF.

**Figure 3:**
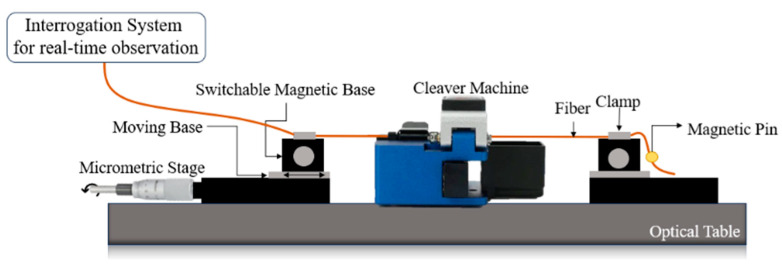
Scheme of the experimental setup for cleavage.

The final step consists of cleaving the CSF. To accurately measure the length of the CSF tip and to optimize the manufacturing process, a translation stage is presented in Figure 3, with an uncertainty of 5 µm associated with the micrometric driver used. The accuracy of the cleavage process is important because the self-image points are accurately positioned throughout the CSF section, and variations of micrometers can lead to the loss of visualization of these points. By achieving the self-image point, the light field is condensed in a particular spatial plane of the CSF section, which results in lower losses. Therefore, the sensitivity of the sensor has improved.

The fiber is clamped between two magnetic bases while ensuring that the SMF-CSF spliced point is aligned with the blade from the cleaver machine. The second clamp secures the end of the fiber between two magnetic pins, ensuring it is under tension. Once the fiber is properly positioned, the micrometric driver is engaged and rotated until the desired length is achieved. As a result, the magnetic base supporting the fiber also moves. The micrometric driver (151-411ME-H, Thorlabs, Newton, NJ, USA) has a travel range of 50 mm, with 10 μm travel per division. It is important to maintain the cleaver machine open before cutting the CSF to facilitate the movement of the fiber.

In the end, several sensors were fabricated with a length of 29.12 mm, and the respective spectrum was observed in real-time in the OSA (Optical Spectrum Analyzer), with wavelengths ranging from 1555 nm to 1590 nm and a full-half width maximum (FHWM) of 12 nm.

### 2.4. Graphene Oxide Coating on Coreless Silica Tip

The final sensor design features one thin layer of GO applied to the CSF tip. GO is a 2D material of carbon atoms packed in a hexagonal lattice, containing carboxyl groups at the margins of its sheets and hydroxyl and epoxy groups within its basal planes [24]. This material offers valuable properties such as solubility in water and organic solvents and the possibility of production on a mass scale. Moreover, the functional groups not only facilitate the effective surface functionalization of the optical fiber but also bestow new abilities upon GO, such as a strong hydrophilic nature as well as increased polymer interaction, among other characteristics. Regarding the presented sensor, the presence of oxygen groups allows for glucose to attach to the film, changing the properties of how light propagates in the sensor region and consequently inducing electromagnetic modulation that is possible to detect. Graphene and its derivatives, such as graphene oxide, can exhibit nonlinear optical behavior, especially at high optical intensities, leading to signal saturation. Depending on its properties and structure, it may exhibit saturation absorption, where at high optical intensities, the absorption saturates and reaches a maximum value. This can impact the transmission of the optical signal through the coated fiber. Finally, GO coatings may also contribute to nonlinear refractive index effects. This can influence the phase of the optical signal and affect its propagation characteristics. These attributes are of great importance in bio-applications as they enable GO to enhance sensor sensitivity [25].

**Figure 4:**
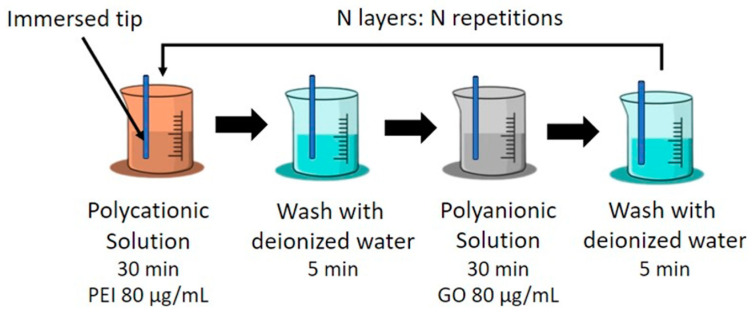
Description of the LbL process.

The Layer-by-Layer (LbL) deposition method, first introduced by J. J. Kirkland and R. K. Iler of DuPont in 1965 [26], serves as the protocol for GO deposition in the presented sensor design. This technique consists of the self-assembly of multilayered thin films through the electrostatic interactions of oppositely charged polyelectrolytes. Moreover, it allows a straightforward and versatile deposition directly on the sensor. Figure 4 illustrates the LbL technique employed for GO deposition, and the protocol is described as follows [27]:

1. The air-dried fiber tip is immersed in a polycationic solution containing polyethyleneimine (PEI, Sigma-Aldrich P3143, Burlington, MA, USA) for 30 min at a concentration of 80 μg/mL. The PEI solution was diluted in a sodium chloride (NaCl) aqueous solution with a concentration of 0.03 g/mL. The inclusion of NaCl results in a more stable solution, which is particularly important when electrostatic interactions are significant.
2. The probe is rinsed in deionized water to remove unbounded PEI and air-dried for 5 min.
3. The tip is immersed in the polyanionic solution of GO (Sigma-Aldrich, 777676, Burlington, MA, USA) with a concentration of 80 μg/mL for 30 min, diluted in deionized water. The GO solution was sonicated for 30 min to reduce agglomeration of the GO flakes, obtaining a higher percentage of monolayer flakes.
4. The probe is rinsed in deionized water to remove unbounded GO and air-dried for 5 min.
5. Repeat step 2.

When using the LbL technique, the uniformity of the GO film coating is dependent on the number of bilayers and on the concentration of the GO solution used [27]. Additionally, the uniformity of the films in the LbL process is dependent on the adsorption properties of the materials used [28]. Naturally, there can be surface irregularities and adhesion variability across the surface of the fiber. Nevertheless, by immersing the fiber in a 1.0 M sodium hydroxide (NaOH) solution prior to the GO deposition process, it is possible to enhance the adhesion of the polycation and polyanion layers. This process removes any biological residues and hydroxylates the surface of the fiber, improving its adhesion properties.

Considering the previous remarks and the results presented by Gongli et al. [29], it is justified to use a concentration of 80 μg/mL for the PEI/GO bilayers.

**Figure 5:**
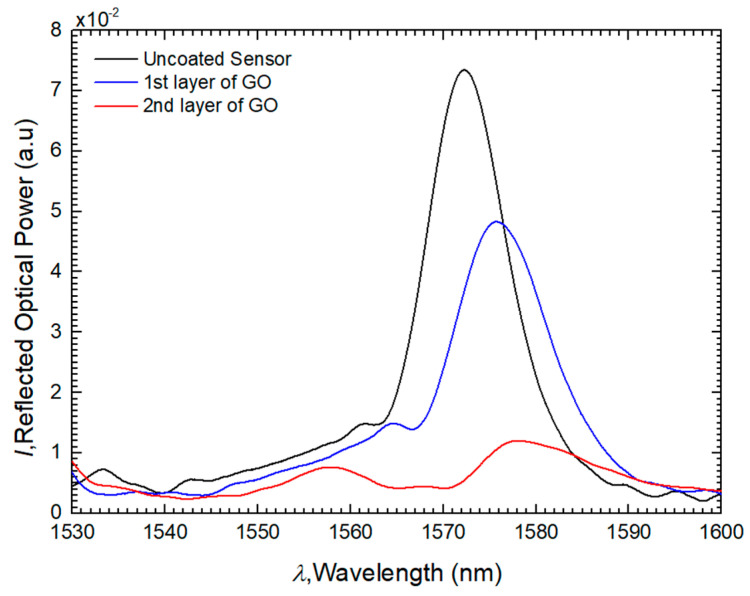
Spectra results for sensor with 2 bilayers of PEI/GO.

Regarding the number of bilayers, a sensor with two bilayers of PEI/GO was fabricated. Figure 5 illustrates the spectra observed in the OSA throughout the LbL process. By adding layers of GO, it is possible to confirm that the spectrum of the sensor undergoes a wavelength redshift and that the reflected optical power is reduced. This phenomenon is attributed to the high refractive index of GO, causing changes in the effective refractive index. Furthermore, it is observed that in the first bilayer of PEI/GO, the characteristic spectrum of the self-image phenomenon is conserved, featuring a well-defined peak. This contrasts with the second bilayer of PEI/GO, where the optical power reduces more drastically, and the previously well-defined peak is no longer preserved. Therefore, the decision was made to adopt the configuration with a single bilayer of PEI/GO.

**Figure 6:**
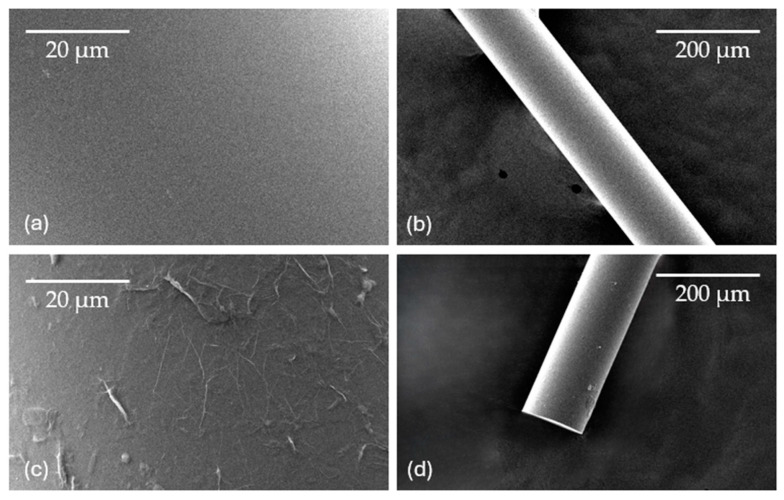
SEM images of (**a**,**b**) uncoated sensors; (**c**,**d**) coated sensors with one bilayer of PEI/GO.

The surface of the sensor, coated with a single bilayer of PEI/GO, was examined using scanning electron microscopy (SEM—Phenom XL from ThermoFisher Scientific, St. Louis, MO, USA). Figure 6a,b depict the surface of the uncoated sensor, while Figure 6c,d showcase the sensor coated with GO. Contrary to the smooth surface of the uncoated sensor, the GO-coated surface exhibits a distinctive wrinkled structure. This structural variation is attributed to the formation of GO nanosheets [30].

### 2.5. Experimental Setup and Methodology

**Figure 7:**
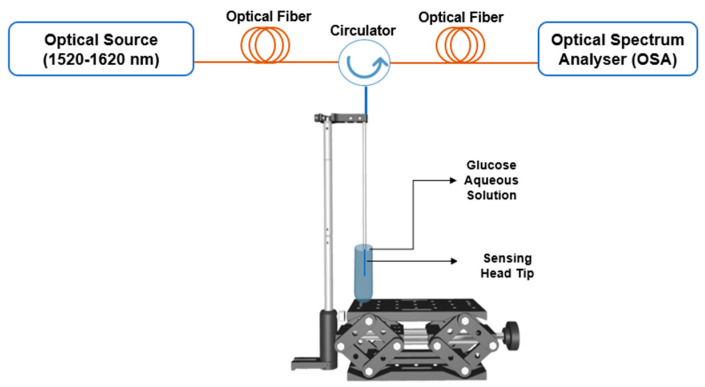
Schematic configuration of the experimental setup.

Figure 7 depicts the experimental setup used in the laboratory, which consisted of a broadband optical source (1520–1620 nm), the sensor, and an OSA (Yokogawa AQ6370C, Tokyo, Japan) connected with a circulator. The OSA has a resolution of 20 pm and can measure wavelengths ranging from 600 to 1700 nm. To ensure stability and to prevent contact between the sensor and the walls of the test sample tubes, the sensor was carefully inserted into a capillary tube. The samples were handled on a lifting platform, which permitted only vertical movement while maintaining the sensor in a fixed position.

Glucose aqueous solutions with concentrations ranging from 25 to 200 mg/dL were prepared through a dilution method in a laboratory-controlled environment at approximately 21 °C. This range was purposely chosen to mimic the glucose concentration levels found in human saliva. A magnetic stirrer (NAHITA, magnetic stirred, model n°690/1) was used to homogenize the aqueous solutions with glucose (G8270-100 g) supplied by Sigma Aldrich. The sensor was immersed in these solutions, and the optical spectra were captured with an OSA. The OSA was configured to operate on a linear scale with a 0.5 nm resolution. For each measurement, the tip was immersed for 150 s before processing the corresponding data.

For the RI characterization of the sensor, the glucose aqueous solutions were prepared in the same manner, and the RI was determined with an Abbe refractometer. The solutions ranged from 0.1 mg/mL to 50 mg/mL, which corresponded to a RI range between 1.3380 RIU and 1.3853 RIU.

## 3. Results

**Figure 8:**
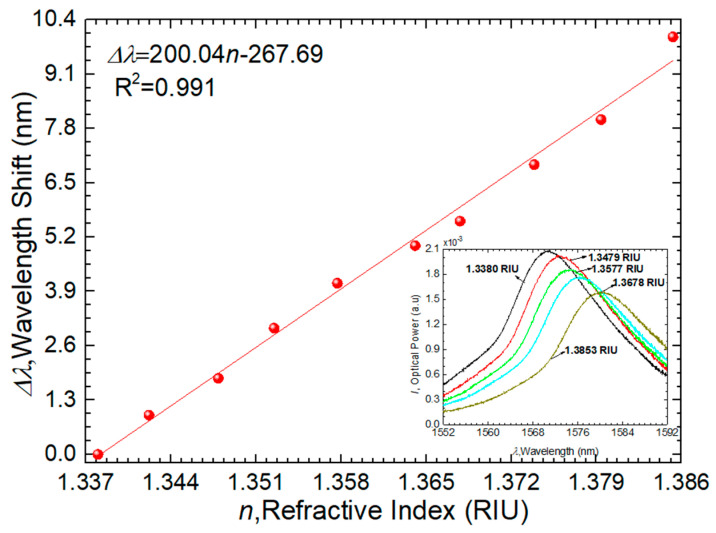
Sensor response to the RI experiment.

Figure 8 presents the results of the GO-based CSF tip response to RI measurements, where the inset graph shows the obtained spectra, described by a predominant maximum peak in the range of (1556–1592) nm.

The experimental results can be described by a linear fitting curve with a correlation coefficient (R^2^) of 0.991. This yields predictable behavior in the response of the sensor regarding RI variations. The total wavelength shift, ∆λ, is equal to 9.9 nm, and the wavelength sensitivity reached a value of 200 ± 6 nm/RIU. Regarding temperature analysis, the glucose sensors operate at room temperature. However, assuming a temperature change of ±10 °C and considering that this type of sensor has a sensitivity of approximately 6.8 pm/°C [13], this temperature variation would correspond to a variation of 68 pm. By studying cross-sensitivity, we can conclude that the proposed sensor revealed high stability with a cross-sensitivity that was determined to be 3.4 × 10^−5^ RIU/°C.

For comparative analysis, we can juxtapose the work conducted by Yan et al. [31], which employs a transmission scheme (SMF + CSF + SMF) with a 29.12 mm length of CSF, a graphene oxide (GO) concentration of 62.5 µg/mL, and a RI range of 1.3333–1.3846 RIU. This study achieved a sensitivity of 172.239 nm/RIU. In contrast, our current work utilizes a typical reflection scheme, yielding a sensitivity of 200 nm/RIU. It is noteworthy that our present work provides a more robust and versatile solution for precise, localized, and straightforward measurements, offering a superior alternative to inline multimode interference sensors. The reflection configuration presented in this study facilitates easier cleaning procedures and allows the use of smaller amounts of analyte, providing a practical advantage in real-world applications.

**Figure 9:**
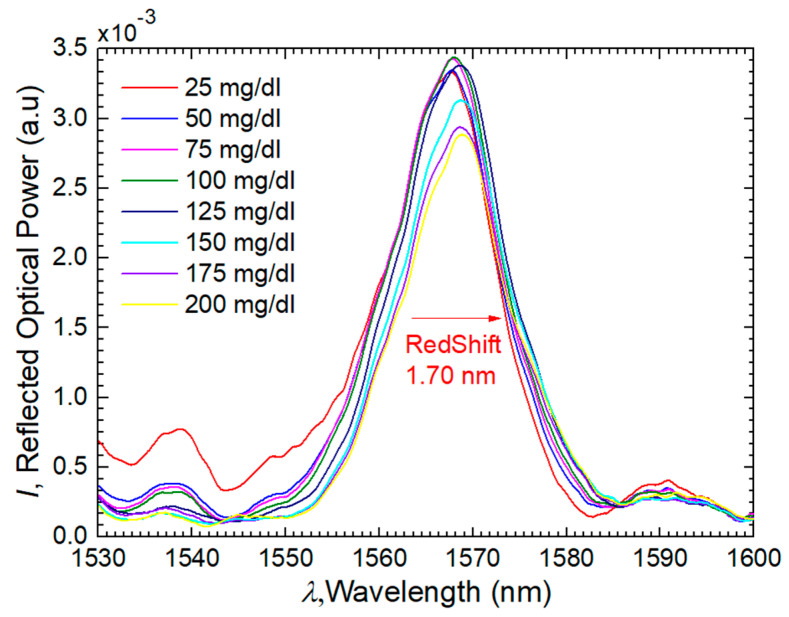
Output spectra of the GO-based CSF tip.

Following the RI characterization, the sensor was tested for glucose in aqueous solutions with concentrations ranging from 25 to 200 mg/dL. Figure 9 presents the experimental spectra, which display variations in optical power and wavelength. The spectra are described by a well-defined peak, as expected from the self-image theory.

**Figure 10:**
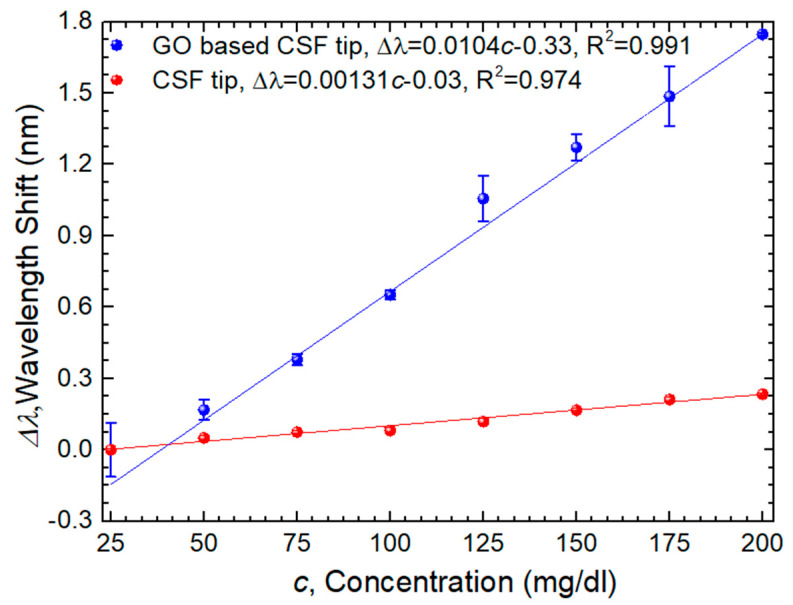
Sensor response to glucose concentration variations.

Figure 10 presents graphically the wavelength response to the different concentrations for the uncoated/coated sensors. The coated sensor shows a ∆λ = 1.70 nm and an R^2^ = 0.991, whereas in the case of the uncoated sensor, the ∆λ is equal to 0.23 nm and the R^2^ is 0.974.

Through the slope of the linear equation, it is possible to determine the wavelength sensitivity of both sensors. In the case of the GO-based CSF tip, it obtained a sensitivity of 10.403 ± 0.004 pm/(mg/dL), while in the CSF tip, it obtained a sensitivity of 1.31 ± 0.08 pm/(mg/dL). This means that by adding one bilayer of PEI/GO with a concentration of 80 µg/mL, the final sensitivity of the optimized sensor increased by a factor of 8.

**Figure 11:**
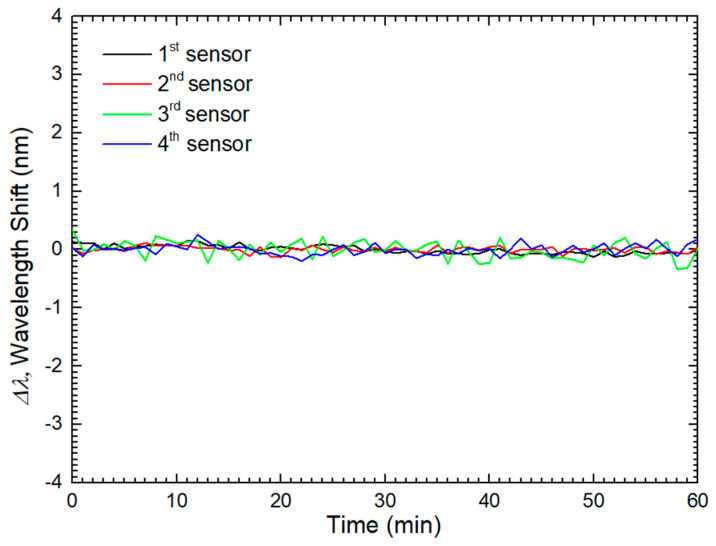
Stability test for different sensors coated with one bilayer of PEI/GO.

To verify if the response of the sensor to glucose-aqueous solutions would be stable over time, a stability test was performed with four tip sensors fabricated in a similar manner. The different sensors were immersed in a glucose-aqueous solution with a concentration of 25 mg/dL. The data were acquired in real-time with an acquisition rate of 1 sample per minute with the OSA. Figure 11 presents the stability results regarding the wavelength variations for the different sensors. It attained an average standard deviation of 0.46 pm/min, meaning that the sensors presented a stable response over time.

The maximum theoretical resolution, $\delta_{t}$, was determined with the following equation [32]:

(2) $$\delta_{t}=\frac{R_{OSA}}{S}$$

where $R_{OSA}$ corresponds to the resolution of the OSA, and S is the sensitivity of the sensor. The GO-coated sensor achieved a resolution of 1.90 mg/dL, meaning that this value corresponds to the maximum theoretical value the sensor can resolve.

It is also possible to determine the limit of detection (*LOD*) of the sensor by taking into consideration the following Equation (3) and assuming a confidence level of 95% [33]:

(3) $$LOD=2\times \frac{\sigma }{S}$$

where σ corresponds to the standard error of interception from Figure 10. Therefore, the final value of *LOD* is 9.15 mg/dL.

Table 3 presents a comparative analysis of the results obtained in this study in relation to other OFS designed for the detection of glucose within a similar concentration range. Notably, all sensors listed in the table, excluding the present work, employ an experimental configuration based on transmission. Despite the apparent higher sensitivities demonstrated by the first two works, the present research contributes a more robust solution by employing a reflection-based system. Firstly, the use of a taper as the sensing area, as seen in [34], renders the sensor more fragile. Secondly, another approach in the table utilizes, beyond graphene oxide, glucose enzymes to enhance sensitivity and specificity, making the sensor more complex and expensive, which is not the focus of study in the present work. As proof of concept, the present sensor based on the reflection configuration has several advantages over the inline configurations. On the one hand, the length is reduced by half since light is reflected at the coreless end face and recoupled to the single-mode fiber. On the other hand, smaller amounts of analyte can be employed, the sensing structure becomes less fragile, and it can be placed at a long distance regarding the acquisition system, thus becoming a good alternative in harsh environments where refractive index or concentration variations need to be monitored.

## 4. Conclusions

This paper introduces a proof of concept using sensors based on self-image theory and GO coating to enhance sensitivity. Prior to the fabrication of the sensor, the theoretical points of the CSF tip self-image were confirmed with Multiphysics COMSOL 6.0 software. Then, the final sensor was fabricated at a length corresponding to the second self-image point, 29.12 mm, with an 80 µg/mL GO film using the LbL technique. The sensor was characterized in RI and achieved a wavelength sensitivity of 200 ± 6 nm/RIU.

Comparative tests of uncoated/coated sensors measuring glucose aqueous solutions ranging from 25 to 200 mg/dL showed an 8-fold increase in sensitivity with the addition of the PEI/GO bilayer and better performance regarding higher values of concentration. The final GO-based sensor achieved a sensitivity of 10.403 ± 0.004 pm/(mg/dL). The sensor also showed a good, stable response with a standard deviation of 0.46 pm/min and a maximum theoretical resolution of 1.90 mg/dL.

To conclude, these findings highlight the enhanced probe sensitivity achieved through self-image theory and GO integration. While numerous RI sensors employing a reflection-based interrogation system have been reported, the present sensor introduces an alternative solution in terms of the testing range for this sensing design since it incorporates self-imaging theory and a graphene oxide coating for sensitive enhancement.

## Figures and Tables

**Table 1:** Self-image points of the CSF for different *p*.

CSF Self-Image Point	
*p*	*L_CSF_* (mm)
1	14.56
2	29.12
3	43.68
4	58.24

**Table 2:** Geometry and RI Settings-CSF (FC125LA) and SMF (SMF28) from Thorlabs.

Settings	Parameter	Value
Geometry	SMF core	8 µm
	SMF cladding	125 µm
	SMF length	5 mm
	CSF diameter	125 µm
	CSF length	14.56 mm for *p* = 1
		29.12 mm for *p* = 2
RI	Operating Wavelength	1550 nm
	SMF core RI	1.4529 RIU
	SMF cladding RI	1.4440 RIU
	CSF RI	1.4440 RIU
	Analyte RI	1.0003 RIU

**Table 3:** Comparison results of glucose sensitivity for different OFS.

Structure	Concentration Range (mg/dL)	Sensitivity (pm/(mg/dL))	Ref.
GOx immobilized SMF microprobe	0–300	17.4	[34]
SMF-TFG (tilted fiber grating)-SMF (GO–GOD)	0–150	13.3	[35]
Fiber SPR & enzymatic reaction device	0–400	5.27	[36]
GO based OFS	0–200	10.403	This work

## Data Availability

Data are contained within the article.
